# Supplementary material for: The adverse effects of bisphosphonates in breast cancer: A systematic review and network meta-analysis
Source: PLoS One. 2021 Feb 5;16(2):e0246441. doi: 10.1371/journal.pone.0246441 (PMC7864400; doi:10.1371/journal.pone.0246441)
Supplement: S1 Fig — Figure showing the trial-specific treatment effects on the event of fever as odds ratios and risk differences, alongside the baseline risks (proportion of the control group who have the event–right hand column). There is wide variability in the reported baseline risks on the right. However, the odds ratios and risk differences are more homogeneous between studies, suggesting that variable recording or reporting quality does not substantially influence the estimates of the treatment effects on risk. The treatment effect on risk is estimated as an odds ratio or risk difference for each study, with an estimate of baseline risk shown in the right panel, and pooled estimates at the bottom. Point estimates and 95% confidence intervals are given–note that some of the upper and lower limits exceed the axis range. In OPTIMIZE-2 there are two estimates, one for each of two bisphosphonate arms. The two pairs of arms in the ABCSG12 trials are shown on separate lines. (PDF) [file pone.0246441.s002.pdf]

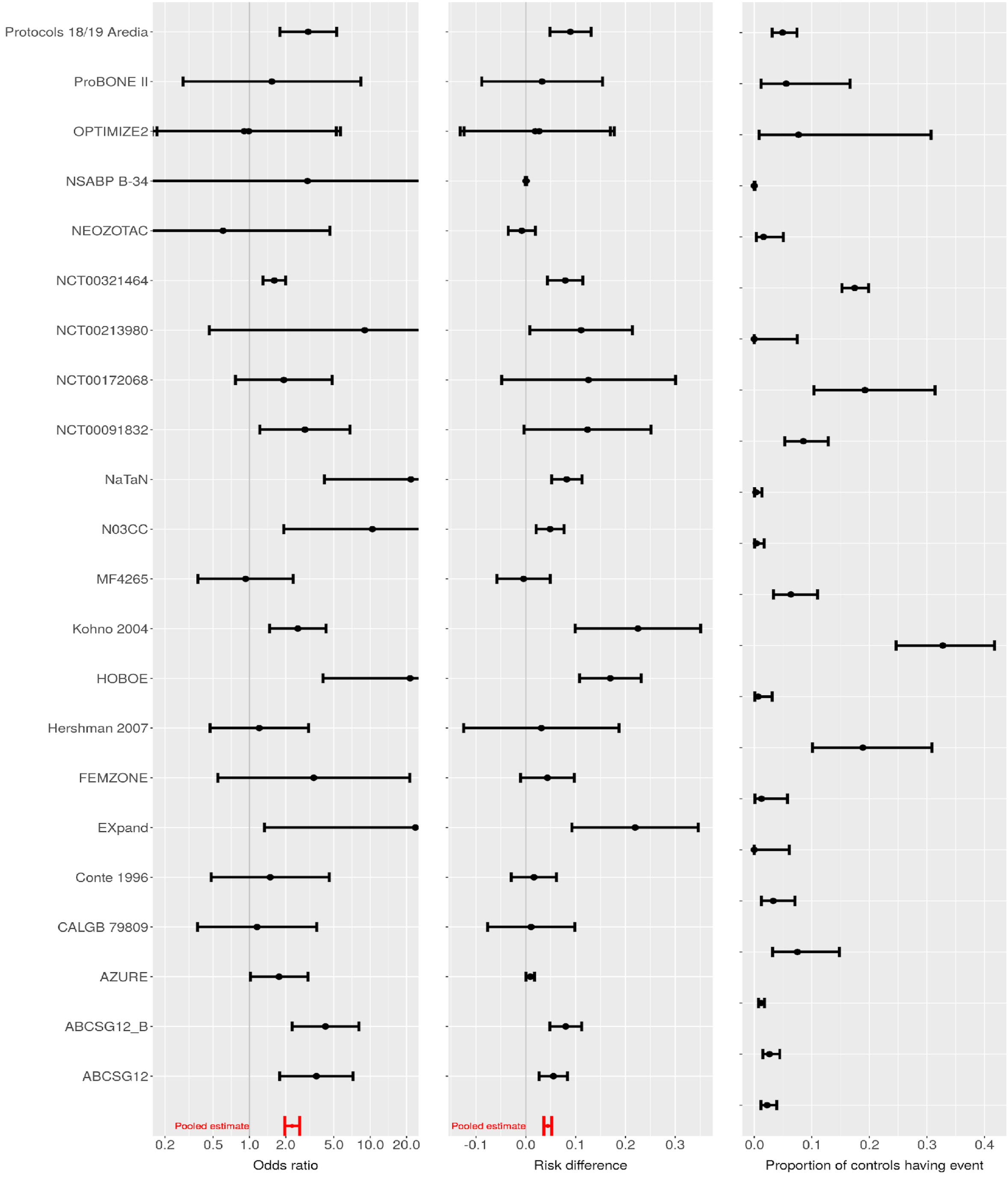

Protocols 18/19 Aredia
ProBONE II
OPTIMIZE2
NSABP B-34
NEOZOTAC
NCT00321464
NCT00213980
NCT00172068
NCT00091832
NaTaN
N03CC
MF4265
Kohno 2004
HOBOE
Hershman 2007
FEMZONE
EXpand
Conte 1996
CALGB 79809
AZURE
ABCSG12_B
ABCSG12
Pooled estimate
0.2
0.5
1.0
2.0
5.0
10.0
20.0
Odds ratio
Pooled estimate
-0.1
0.0
0.1
0.2
0.3
Risk difference
0.0
0.1
0.2
0.3
0.4
Proportion of controls having event
