# Supplementary material for: The adverse effects of bisphosphonates in breast cancer: A systematic review and network meta-analysis
Source: PLoS One. 2021 Feb 5;16(2):e0246441. doi: 10.1371/journal.pone.0246441 (PMC7864400; doi:10.1371/journal.pone.0246441)
Supplement: S1 Table — (DOCX) [file pone.0246441.s003.docx]

# The adverse effects of bisphosphonates as adjuvant therapy in breast cancer: a piggy-back systematic review and network meta-analysis

Christopher Jackson, Alexandra LJ Freeman, Zśofia Szlamka, David J Spiegelhalter

## Supporting Information

**S1 Table 1: Trials included in the analysis and their characteristics**

| **Trial name**  **(countries)** | **Total n per protocol (intention to treat)** | **Average ages in each arm**  **Metastatic status** | **Bisphosphonate comparison** | **Concurrent therapies** | **Blinding** | **Completeness data recording** |
| --- | --- | --- | --- | --- | --- | --- |
| Conte 1996 [1]  (Argentina, Canada, France, Italy, Spain, Switzerland, UK) | 294  (295) | not reported  All had bone metastases | iv pamidronate versus observation only | Chemotherapy (CMF, CEF or CAF) | open label | Very limited |
| ARIBON [2]  N0276137347  (UK) | 48  (50) | Medians 67.8, 67.5  No distant metastases | Oral ibandronate versus oral placebo | Anastrozole | double blinded | Very limited |
| ZICE [3]  NCT00326820  (UK) | 1326  (1401) | Medians 61, 61  All had bone metastases | Oral ibandronate versus iv zoledronic acid | Chemotherapy, hormone therapy & radiotherapy | open label | >=10% frequency threshold |
| MF4265 [4]  (Europe, Kuwait, Russia, South  Africa and the United States) | 463  (466) | Medians 53, 55.5, 57  All had bone metastases | iv ibandronate (2mg) versus iv ibandronate (6mg) versus iv placebo | Any allowed | double blinded | Limited (no stated reporting threshold) |
| Von Au 2016 [5]  (Germany) | 321  (375) | ‘Average’ 53, 52.8, 52.9  All had bone metastases | iv pamidronate versus iv clodronate versus oral clodronate | Not clear | open label | very limited |
| Rebbeca II [6]  NCT00485953  (US) | 109  (109) | Means 64.7, 63.8  Not reported | Oral risedronate versus oral placebo | Aromatase inhibitors | double blinded | very limited |
| Kristensen 2008 [7]  (Denmark, Sweden, Iceland) | 884  (953) | Not given as average  No distant metastases | Oral pamidronate versus observation only | CEF or CMF chemotherapy (reported separately). Radiotherapy allowed, but no hormone therapy | open label | very limited |
| NSABP B-34  [8, 9]  NCT00009945  (North America) | 3235  (3323) | Means 54, 53  No distant metastases | Oral clodronate versus oral placebo | Any allowed | double blinded | all serious adverse events, >=5% threshold for non-serious |
| Hershman 2007 [10]  (US) | 103  (114) | Means 42, 43  No distant metastases | iv zoledronic acid versus iv placebo | Chemotherapy  No other treatments | double blinded | Limited (no stated reporting threshold) |
| ‘Z-FAST Japan’ [11]  UMIN000001104  (Japan) | 192  (204) | Medians 60, 60  No distant metastases | iv zoledronic acid versus ‘delayed’ iv zoledronic acid | Letrozole  Chemotherapy also allowed | open label | >=5% reporting threshold |
| ABCSG-12 [12]  NCT00295646  (Austria) | (1803) | medians 45.5, 45.3, 45, 44.5  No distant metastases | iv zoledronic acid versus observation only | Goserelin & tamoxifen or goserelin & anastrozole (reported separately)  No chemotherapy (though some had had neoadjuvant chemotherapy) | open label | >=10% reporting threshold |
| SABRE [13]  NCT00082277  (US, Canada, France, Greece, Netherlands, S Africa, Spain, UK) | 154  (154) | means 64.8, 63.8  No distant metastases | Oral risedronate versus oral placebo | Anastrozole.  No chemotherapy | double blinded | All recorded adverse events reported |
| Kohno 2004 [14]  (Japan) | 227  (228) | Means 54.3, 53.5  All had bone metastases | iv zoledronic acid versus iv placebo | Chemotherapy and/or hormone therapy allowed | double blinded | >=15% reporting threshold |
| OPTIMIZE-2 [15]  NCT00320710  (United States) | 413  (416) | Means 58.6, 60.8  All had bone metastases | iv zoledronic acid (every 3 weeks) versus iv zoledronic acid (every 12 weeks) versus iv placebo | Any other therapy permitted | double blinded | all serious adverse events, >=5% threshold for non-serious |
| Bonadiuv [16]  NCT02616744  (Italy) | 171  (202) | Medians 59.6, 60.5  No distant metastases | Oral ibandronate versus placebo | Aromatase inhibitors | single blinded | Very limited |
| Paterson 1993 [17]  (UK, Canada) | (173) | Medians 58, 61  All had bone metastases | Oral clodronate versus placebo | Chemotherapy and/or hormone therapy allowed | double blinded | All recorded adverse events (deemed related to drug) reported |
| CALGB trial 79809 [18]  NCT00022087  (US) | (150) | Medians 46, 48  No distant metastases | iv zoledronic acid versus observation only | Chemotherapy | open label | Very limited |
| Macpherson 2015 [19]  (UK) | 29  (34) | Medians 60, 61  All had bone metastases | iv ibandronate followed by oral ibandronate versus oral ibandronate only | Hormone therapy  Chemotherapy and radiotherapy also allowed | open label | All adverse events over grade 2 reported |
| ISRCTN83688026 [20–22]  (UK, Canada, Norway, Finland) | 1069  (1079) | Means 52.8, 52.7  All had bone metastases | Oral clodronate versus oral placebo | Chemotherapy and/or hormone therapy allowed | double blinded | All recorded, but limited reporting |
| ProBONE I [23]  NCT00333229  (Germany) | 11  (11) | Means 41.2, 43.2  No distant metastases | iv zoledronic acid versus iv placebo | Not specified | double blinded | all serious adverse events, >=5% threshold for non-serious |
| ProBONE II [24, 25]  NCT00375505  (Germany) | 70  (70) | Means 42.8, 43.2  No distant metastases | iv zoledronic acid versus iv placebo | Chemotherapy and/or hormone therapy | double blinded | >10% reporting threshold |
| NCT00091832  [26–28]  (US) | (254) | Means 52, 57.8  All had bone metastases | iv ‘bisphosphonates’ (1 pamidronate, the rest zoledronic acid) versus iv denosumab | Chemotherapy and/or hormone therapy | single blinded | >10% reporting threshold |
| NCT00321464  [29]  (“322 centres in  Europe, North America, South America, Japan, Australia, India, and South  Africa”) | 2033  (2046) | Means 56.6, 56.8  All had bone metastases | iv zoledronic acid versus iv denosumab | Any other therapy allowed | double blinded | all serious adverse events, >=5% threshold for non-serious |
| MF 4434  MF 4414 [30, 31]  (Europe, Australia, New Zealand, Kuwait, Russia, S Africa, Bulgaria, US) | 845 | Medians 56, 57 (20mg arm: age not reported)  All had bone metastases | Oral ibandronate (50mg) versus oral ibandronate (20mg) versus oral placebo | Any other therapy allowed | double blinded | >=2% reporting threshold (‘treatment-related’ only) |
| AZURE [32]  BIG 01-04  NCT00072020,  ISRCTN79831382  ‘seven countries’ | 3340  (3360) | Means 51.3, 51.6  No distant metastases | iv zoledronic acid versus observation only | Chemotherapy and/or hormone therapy allowed | open label | >10% reporting threshold  Full individual data provided by authors |
| Rosen 2003 [33]  (Canada, US, Argentina, Brazil, Chile, Peru, Uruguay, Austria, Belgium, Czech Republic, France, Germany, Ireland, Italy, Netherlands, Poland, Spain, Sweden, Switzerland, UK, Australia, Israel, New Zealand, S Africa) | (1130) | Medians 58, 58, 56  All had bone metastases | iv zoledronic acid (4mg) versus iv zoledronic acid (8/4mg) versus pamidronate (90mg) | Chemotherapy and/or hormone therapy allowed | open label | >=15% reporting threshold |
| ZOOM [34]  NCT00375427  EudraCT, number 2005-004942-15  (Italy) | 425  (425) | Means 60.4, 59.8  All had bone metastases | iv zoledronic acid (every 3 months) versus iv zoledronic acid (every 4 weeks) after 1 year on za already | Any other therapy allowed | open label | >=1% threshold for serious events, >=5% threshold for non-serious |
| Delmas 1997 [35]  (France?) | 53  (53) | Means 46.6-45.7  No mention of metastases | Oral risedronate versus oral placebo | Not mentioned | double blinded | Very limited |
| Rotstein 1992 [36]  (Sweden, Denmark) | 44  (44) | Medians 51, 55  All had bone metastases | iv clodronate versus iv placebo | Chemotherapy and/or hormone therapy allowed.  No radiotherapy | double blinded | Very limited |
| NaTaN [37]  GBG 36/ABCSG 29  NCT00512993  (Germany, Austria) | 674  (693) | Averages not reported  No distant metastases | iv zoledronic acid versus observation only | Any other therapy allowed | open label | All adverse events in predefined categories reported |
| GAIN [38]  NCT00196872  (Germany) | 2800  (2994) | Averages not reported  No distant metastases | Oral ibandronate versus observation only | Specific chemotherapy regimes specified.  Radiotherapy, hormone therapy and/or trastuzumab allowed | open label | Adverse events reported only by organ class |
| N03CC [39]  Alliance  NCT00107263  (US) | 541  (558) | Means 59.2, 59.6  No distant metastases | iv zoledronic acid versus ‘delayed’ zoledronic acid – this second group treated as an ‘observation only’ control group because data of adverse events from only the first 6 months used, before the ‘delayed’ group were given any zoledronic acid. | Letrozole  No other anticancer therapy allowed | open label | >=4% reporting threshold |
| ZO-FAST  [40, 41]  CFEM345D2405  NCT00171340  (Argentina, Australia, Belgium, Brazil, Chile, China, Colombia, Czech Republic. Egypt, Finland, France, Germany, Guatemala, Hong Kong, Italy, S Korea, Mexico, Netherlands, New Zealand, Peru, Philippines, Portugal, Spain, Switzerland, Taiwan, Thailand, UK, Venezuela) | 1060  (1065) | Medians 57, 58  No distant metastases | iv zoledronic acid versus ‘delayed’ iv zoledronic acid | Letrozole  No other anti-cancer therapy allowed | open label | all serious adverse events, >=5% threshold for non-serious |
| E-ZO-FAST  [42, 43]  CFEM345D2406  NCT00171314  (Argentina, Belgium, France, Italy, S Korea, Netherlands, Saudi Arabia, S Africa, Spain) | 523  (527) | Means 58.2, 58.4  No distant metastases | iv zoledronic acid versus ‘delayed’ zoledronic acid | Letrozole  No other anti-cancer therapies allowed | open label | all serious adverse events, >=5% threshold for non-serious |
| Z-FAST [44]  CZOL446EUS32  NCT00050011  (US, Puerto Rico) | 600  (600) | Means 61.4, 61.0  No distant metastases | iv zoledronic acid versus ‘delayed’ zoledronic acid | Letrozole  No other anti-cancer therapies allowed | open label | all serious adverse events, >=5% threshold for non-serious |
| Pivot 2011 [45]  (France) | 312  (325) | Medians 59, 60  All had bone metastases | iv ibandronate (15 min infusion) versus iv ibandronate (60 min infusion) | Chemotherapy | open label | Very limited |
| NCT00376740  [46]  (Israel) | 86  (90) | Medians 59.3, 58  No distant metastases | iv zoledronic acid versus observation only | Letrozole  (all had previously had tamoxifen) | open label | ‘Most common’ only listed |
| HOBOE [47]  NCT00412022  (Italy) | 453  (483) | Medians 50, 49  No distant metastases | iv zoledronic acid versus observation only | Letrozole  Radiotherapy and triptorelin allowed | open label | All recorded adverse events reported |
| Body 2007 [48]  (‘seven countries’) | 274  (275) | Medians 55, 55  All had bone metastases | Oral ibandronate versus iv zoledronic acid | Any other treatment allowed | open label | Very limited |
| ML17632 [49]  (Austria, Switzerland, Germany) | 127  (132) | Means 61, 60  All had bone metastases | iv ibandronate (15 min infusions) versus iv ibandronate (60min infusions) | Any other treatment allowed | open label | >10% reporting threshold |
| N02C1 [50]  NCT00054418  (US) | 212  (216) | Means 43.3, 43.6  No distant metastases | Oral risendronate versus oral placebo | Any other treatment allowed | double blinded | >3% reporting threshold |
| Saarto 2008 [51]  Finland | 282  (299) | Means 52, 52  No distant metastases | Oral clodronate versus observation only | Radiotherapy, chemotherapy and/or hormone therapy allowed | open label | full data shared by author |
| Protocol 18/19 Aredia Breast Cancer Study Group [52]  (US, Canada, Australia, New Zealand) | 753  (754) | Averages not reported  All had bone metastases | iv pamidronate versus iv placebo | Chemotherapy and/or hormone therapy allowed | double blinded | Very limited |
| Coleman 1998 [53]  (UK, Denmark) | 47  (47) | Overall median 53  All had bone metastases | Oral pamidronate (150mg) versus oral pamidronate (300mg) | Hormone therapy and radiotherapy allowed.  No chemotherapy | double blinded | All adverse effects ‘at least possibly related to’ study drug reported |
| NCT00213980  [54, 55]  (US) | 68  (68) | Means 50.5, 54.5  No distant metastases | iv zoledronic acid versus observation only | All other therapies allowed | open label | >=5% reporting threshold |
| NCT00172068  [56]  (Germany) | 96  (96) | Medians 54, 54  No distant metastases | iv zoledronic acid versus observation only | Any other therapy allowed | open label | >=20% reporting threshold |
| EXPAND [57, 58]  CFEM345DDE09  NCT00332709  (Germany) | 81  (83) | Means 61.3, 58.4  No distant metastases | iv zoledronic acid versus observation only | Letrozole  No other treatments mentioned | open label | all serious adverse events, >=5% threshold for non-serious |
| REFORM [59]  (Canada) | 38  (38) | Medians 50, 60  All had bone metastases | iv pamidronate (every 4 weeks) versus iv pamidronate (every 12 weeks) | All other therapies permitted | open label | Very limited |
| ODYSSEY [60]  NCT01907880  (Canada) | 73  (73) | Medians 62.9, 61.3  All had bone metastases | iv zoledronic acid versus iv pamidronate | Previous iv pamidronate therapy  Any other treatment allowed concurrently | double blinded | Limited |
| S0307 [61]  NCT00127205  (US) | 5800  (6097) | Means 53, 52.6, 52.7  No distant metastases | iv zoledronic acid  versus oral clodronate  versus oral ibandronate | Any other treatment allowed concurrently | open label | all serious adverse events, >=5% threshold for non-serious |
| NEOZOTAC [62]  NCT01099436  (Netherlands) | 246  (250) | Means 48.9, 49.5  No distant metastases | Neoadjuvant  iv zoledronic acid versus observation only | TAC chemotherapy  No hormone therapy or trastuzumab | open label | All grade 3+ events recorded, reported. |
| NEOZOL [63]  NCT01367288  (France) | 50  (53) | Means 51.2, 50.5  No distant metastases | Neoadjuvant  iv zoledronic acid versus observation only | Chemotherapy  Trastuzumab allowed, but no hormone therapy | open label | Very limited |
| JONIE [64]  UMIN000003261  (Japan) | 186  (188) | Means 49.5, 49  No distant metastases | Neoadjuvant  iv zoledronic acid versus observation only | 4 cycles of FEC100 followed by 12 weekly cycles of paclitaxel | open label | All grade 3+ recorded, reported. |
| FEMZONE [65]  EUCTR2004- 004007-37-DE  NCT00375752  (Germany) | 168  (168) | Means 70.5, 71.3  No distant metastases | Neoadjuvant  iv zoledronic acid versus observation only | Letrozole  No other therapies | open label | all serious adverse events, >=5% threshold for non-serious |
| NEOCAN [66]  NCT00247650  (Canada) | 10  (10) | Medians 78, 67  No distant metastases | Neoadjuvant  iv zoledronic acid versus observation only | Letrozole  No other therapies | open label | ‘most frequent’ adverse events reported |
| NCT00242203  [67]  (US) | 119  (120) | Medians 49. 47  No distant metastases | Neoadjuvant  iv zoledronic acid versus observation only | Chemotherapy: epirubicin plus docetaxel every 3 weeks | open label | All grade 3+ recorded, reported. |

## Supplementary Material:

## References

1. Conte PF, Latreille J, Mauriac L, Calabresi F, Santos R, Campos D, Bonneterre J, Francini G, Ford JM (1996) Delay in progression of bone metastases in breast cancer patients treated with intravenous pamidronate: Results from a multinational randomized controlled trial. J Clin Oncol 14:2552–2559

2. Lester JE, Dodwell D, Purohit OP, Gutcher SA, Ellis SP, Thorpe R, Horsman JM, Brown JE, Hannon RA, Coleman RE (2008) Prevention of anastrozole-induced bone loss with monthly oral ibandronate during adjuvant aromatase inhibitor therapy for breast cancer. Clin Cancer Res 14:6336–6342

3. Barrett-Lee P, Casbard A, Abraham J, et al (2014) Oral ibandronic acid versus intravenous zoledronic acid in treatment of bone metastases from breast cancer: A randomised, open label, non-inferiority phase 3 trial. Lancet Oncol 15:114–122

4. Diel IJ (2004) Ibandronate: A well-tolerated intravenous and oral treatment for metastatic bone disease. Eur J Cancer, Suppl 2:13–16

5. von Au A, Milloth E, Diel I, et al (2016) Intravenous pamidronate versus oral and intravenous clodronate in bone metastatic breast cancer: A randomized, open-label, non-inferiority phase III trial. Onco Targets Ther 9:4173–4180

6. Greenspan SL, Vujevich KT, Brufsky A, Lembersky BC, Londen GJ van, Jankowitz RC, Puhalla SL, Rastogi P, Perera S (2015) Prevention of Bone Loss with Risedronate in Breast Cancer Survivors: A Randomized, Controlled Clinical Trial. Osteoporos Int 26:1857–1864

7. Kristensen B, Ejlertsen B, Mouridsen HT, et al (2008) Bisphosphonate treatment in primary breast cancer: Results from a randomised comparison of oral pamidronate versus no pamidronate in patients with primary breast cancer. Acta Oncol (Madr) 47:740–746

8. Paterson AHG, Anderson SJ, Lembersky BC, et al (2012) Oral clodronate for adjuvant treatment of operable breast cancer (National Surgical Adjuvant Breast and Bowel Project protocol B-34): A multicentre, placebo-controlled, randomised trial. Lancet Oncol 13:734–742

9. ClinicalTrials.gov Clodronate With or Without Chemotherapy and/or Hormonal Therapy in Treating Women With Stage I or Stage II Breast Cancer.

10. Hershman DL, McMahon DJ, Crew KD, Cremers S, Irani D, Cucchiara G, Brafman L, Shane E (2008) Zoledronic acid prevents bone loss in premenopausal women undergoing adjuvant chemotherapy for early-stage breast cancer. J Clin Oncol 26:4739–4745

11. Takahashi S, Iwase T, Kohno N, et al (2012) Efficacy of zoledronic acid in postmenopausal Japanese women with early breast cancer receiving adjuvant letrozole: 12-month results. Breast Cancer Res Treat 133:685–693

12. Gnant M, Mlineritsch B, Schippinger W, et al (2009) Endocrine therapy plus zoledronic acid in premenopausal breast cancer. N Engl J Med 360:679–691

13. Van Poznak C, Hannon RA, Mackey JR, Campone M, Apffelstaedt JP, Clack G, Barlow D, Makris A, Eastell R (2010) Prevention of aromatase inhibitor-induced bone loss using risedronate: The SABRE trial. J Clin Oncol 28:967–975

14. Kohno N, Aogi K, Minami H, Nakamura S, Asaga T, Iino Y, Watanabe T, Goessl C, Ohashi Y, Takashima S (2005) Zoledronic acid significantly reduces skeletal complications compared with placebo in Japanese women with bone metastases from breast cancer: A randomized, placebo-controlled trial. J Clin Oncol 23:3314–3321

15. ClinicalTrials.gov Continued Efficacy and Safety of Zoledronic Acid (q 4 Wks vs. q 12 Wks) in the 2nd Year of Treatment in Patients With Bone Metastases From Breast Cancer.

16. Livi L, Meattini I, Scotti V, Saieva C, Desideri I, Carta GA (2016) BONADIUV trial: A single blind, randomized placebo controlled phase II study using oral ibandronate for osteopenic women receiving adjuvant aromatase inhibitors: Final safety analysis. J Clin Oncol 34:e12043

17. Paterson AH, Powles TJ, Kanis JA, McCloskey E, Hanson J, Ashley S (1993) Double-blind trial of oral clodronate in breast cancer patients with bone metastases. Bull Cancer 80:50–56

18. Shapiro CL, Halabi S, Hars V, et al (2011) Zoledronic acid preserves bone mineral density in premenopausal women who develop ovarian failure due to adjuvant chemotherapy: Final results from CALGB trial 79809. Eur J Cancer 47:683–689

19. MacPherson IR, Bray C, Hopkins C, Hannon RA, Lewsley LA, Ritchie DM, Canney P (2015) Loading dose ibandronate versus standard oral ibandronate in patients with bone metastases from breast cancer. Clin Breast Cancer 15:117–127

20. Powles T, Paterson A, McCloskey E, Schein P, Scheffler B, Tidy A, Ashley S, Smith I, Ottestad L, Kanis J (2006) Reduction in bone relapse and improved survival with oral clodronate for adjuvant treatment of operable breast cancer [ISRCTN83688026]. Breast Cancer Res 8:1–7

21. Atula S, Powles T, Paterson A, McCloskey E, Nevalainen J, Kanis J (2003) Extended safety profile of oral clodronate after long-term use in primary breast cancer patients. Drug Saf 26:661–671

22. Powles T, Paterson S, Kanis JA, et al (2002) Randomized, placebo-controlled trial of clodronate in patients with primary operable breast cancer. J Clin Oncol 20:3219–3224

23. ClinicalTrials.gov A Study of Zoledronic Acid in the Prevention of Cancer Therapy-induced Bone Loss.

24. ClinicalTrials.gov The Safety and Efficacy of Zoledronic Acid in the Prevention of Cancer Therapy Induced Bone Loss.

25. Hadji P, Kauka A, Ziller M, Birkholz K, Baier M, Muth M, Bauer M (2014) Effects of zoledronic acid on bone mineral density in premenopausal women receiving neoadjuvant or adjuvant therapies for HR+ breast cancer: The ProBONE II study. Osteoporos Int 25:1369–1378

26. Lipton A, Steger GG, Figueroa J, et al (2007) Randomized active-controlled phase II study of denosumab efficacy and safety in patients with breast cancer-related bone metastases. J Clin Oncol 25:4431–4437

27. Campbell-Baird C, Lipton A, Sarkeshik M, Ma H, Jun S (2010) Incidence of acute phase adverse events following denosumab or intravenous bisphosphonates: Results from a randomized, controlled phase II study in patients with breast cancer and bone metastases. Community Oncol 7:85–89

28. Lipton A, Steger GG, Figueroa J, et al (2008) Extended efficacy and safety of denosumab in breast cancer patients with bone metastases not receiving prior bisphosphonate therapy. Clin Cancer Res 14:6690–6696

29. ClinicalTrials.gov A Study to Compare Denosumab With Zoledronic Acid in Subjects With Bone Metastases From Solid Tumors.

30. Body JJ, Diel IJ, Liehinitzer M, Lazarev A, Pecherstorfer M, Bell R, Tripathy D, Bergstrom B (2004) Oral ibandronate reduces the risk of skeletal complications in breast cancer patients with metastatic bone disease: Results from two randomised, placebo-controlled phase III studies. Br J Cancer 90:1133–1137

31. EMEA (2004) Report on scientific discussion of Bondronat.

32. Coleman R, Woodward E, Brown J, et al (2011) Safety of zoledronic acid and incidence of osteonecrosis of the jaw (ONJ) during adjuvant therapy in a randomised phase III trial (AZURE: BIG 01-04) for women with stage II/III breast cancer. Breast Cancer Res Treat 127:429–438

33. Rosen LS, Gordon DH, Dugan W, et al (2004) Zoledronic Acid Is Superior to Pamidronate for the Treatment of Bone Metastases in Breast Carcinoma Patients with at Least One Osteolytic Lesion. Cancer 100:36–43

34. Amadori D, Aglietta M, Alessi B, et al (2013) Efficacy and safety of 12-weekly versus 4-weekly zoledronic acid for prolonged treatment of patients with bone metastases from breast cancer (ZOOM): A phase 3, open-label, randomised, non-inferiority trial. Lancet Oncol 14:663–670

35. Delmas PD, Balena R, Confravreux E, Hardouin C, Hardy P, Bremond A (1997) Bisphosphonate risedronate prevents bone loss in women with artificial menopause due to chemotherapy of breast cancer: A double-blind, placebo- controlled study. J Clin Oncol 15:955–962

36. Rotstein S, Glas U, Eriksson M, Pfeiffer P, Hansen J, Söderqvist J, Bandmann U, Strid S (1992) Intravenous clodronate for the treatment of hypercalcaemia in breast cancer patients with bone metastases-A prospective randomised placebo-controlled multicentre study. Eur J Cancer 28:890–893

37. Von Minckwitz G, Rezai M, Tesch H, et al (2016) Zoledronate for patients with invasive residual disease after anthracyclines-taxane-based chemotherapy for early breast cancer - The Phase III NeoAdjuvant Trial Add-oN (NaTaN) study (GBG 36/ABCSG 29). Eur J Cancer 64:12–21

38. Von Minckwitz G, Möbus V, Schneeweiss A, et al (2013) German adjuvant intergroup node-positive study: A phase III trial to compare oral ibandronate versus observation in patients with high-risk early breast cancer. J Clin Oncol 31:3531–3539

39. Hines SL, Mincey B, Dentchev T, et al (2009) Immediate versus delayed zoledronic acid for prevention of bone loss in postmenopausal women with breast cancer starting letrozole after tamoxifen-N03CC. Breast Cancer Res Treat 117:603–609

40. Coleman R, De Boer R, Eidtmann H, et al (2013) Zoledronic acid (zoledronate) for postmenopausal women with early breast cancer receiving adjuvant letrozole (ZO-FAST study): Final 60-month results. Ann Oncol 24:398–405

41. ClinicalTrials.gov Zoledronic Acid in the Prevention of Cancer Treatment Related Bone Loss in Postmenopausal Women Receiving Letrozole for Breast Cancer.

42. Llombart A, Frassoldati A, Paija O, Sleeboom HP, Jerusalem G, Mebis J, Deleu I, Miller J, Schenk N, Neven P (2012) Immediate administration of zoledronic acid reduces aromatase inhibitorassociated bone loss in postmenopausal women with early breast cancer: 12-month analysis of the E-ZO-FAST trial. Clin Breast Cancer 12:40–48

43. ClinicalTrials.gov The Use of Zoledronic Acid to Prevent Cancer-treatment Bone Loss in Post-menopausal Women Receiving Adjuvant Letrozole for Breast Cancer.

44. Brufsky AM, Bosserman LD, Caradonna RR, Haley BB, Jones CM, Moore HCF, Jin L, Warsi GM, Ericson SG, Perez EA (2009) Zoledronic acid effectively prevents aromatase inhibitor- associated bone loss in postmenopausal women with early breast cancer receiving adjuvant letrozole: Z-fast study 36-month follow-up results. Clin Breast Cancer 9:77–85

45. Pivot X, Lortholary A, Abadie-Lacourtoisie S, Mefti-Lacheraf F, Pujade-Lauraine E, Lefeuvre C, Letessier S, Morvan P, Dür C, Frimat L (2011) Renal safety of ibandronate 6 mg infused over 15 min versus 60 min in breast cancer patients with bone metastases: A randomized open-label equivalence trial. Breast 20:510–514

46. Safra T, Bernstein-Molho R, Greenberg J, Pelles-Avraham S, Stephansky I, Sarid D, Inbar MJ, Stemmer SM, Geffen DB (2012) The protective effect of zoledronic acid on bone loss in postmenopausal women with early breast cancer treated with sequential tamoxifen and letrozole: A prospective, randomized, phase II trial. Oncology 81:298–305

47. Nuzzo F, Gallo C, Lastoria S, et al (2012) Bone effect of adjuvant tamoxifen, letrozole or letrozole plus zoledronic acid in early-stage breast cancer: The randomized phase 3 hoboe study. Ann Oncol 23:2027–2033

48. Body JJ, Lichinitser M, Tjulandin S, Garnero P, Bergström B (2007) Oral ibandronate is as active as intravenous zoledronic acid for reducing bone turnover markers in women with breast cancer and bone metastases. Ann Oncol 18:1165–1171

49. von Moos R, Caspar CB, Thürlimann B, Angst R, Inauen R, Greil R, Bergstrom B, Schmieding K, Pecherstorfer M (2008) Renal safety profiles of ibandronate 6 mg infused over 15 and 60 min: A randomized, open-label study. Ann Oncol 19:1266–1270

50. Hines SL, Mincey BA, Sloan JA, Thomas SP, Chottiner E, Loprinzi CL, Carlson MD, Atherton PJ, Salim M, Perez EA (2009) Phase III randomized, placebo-controlled, double-blind trial of risedronate for the prevention of bone loss in premenopausal women undergoing chemotherapy for primary breast cancer. J Clin Oncol 27:1047–1053

51. Saarto T, Blomqvist C, Virkkunen P, Elomaa I (2001) Adjuvant clodronate treatment does not reduce the frequency of skeletal metastases in node-positive breast cancer patients: 5-Year results of a randomized controlled trial. J Clin Oncol 19:10–17

52. Lipton A, Theriault RL, Hortobagyi GN, Simeone J, Knight RD, Mellars K, Reitsma DJ, Heffernan M, Seaman JJ (2000) Pamidronate prevents skeletal complications and is effective palliative treatment in women with breast carcinoma and osteolytic bone metastases: Long term follow-up of two randomized, placebo-controlled trials. Cancer 88:1082–1090

53. Coleman RE, Houston S, Purohit OP, Rubens RD, Kandra A, Ford J (1998) A randomised phase II study of oral pamidronate for the treatment of bone metastases from breast cancer. Eur J Cancer 34:820–824

54. Leal T, Tevaarwerk A, Love R, Stewart J, Binkley N, Eickhoff J, Parrot B, Mulkerin D (2010) Randomized trial of adjuvant zoledronic acid in postmenopausal women with high-risk breast cancer. Clin Breast Cancer 10:471–476

55. ClinicalTrials.gov Bone Mineral Density Effects of Zoledronate in Postmenopausal Women With Breast Cancer.

56. Solomayer EF, Gebauer G, Hirnle P, et al (2012) Influence of zoledronic acid on disseminated tumor cells in primary breast cancer patients. Ann Oncol 23:2271–2277

57. ClinicalTrials.gov Safety/Efficacy of Letrozole Monotherapy or in Combination With Zoledronic Acid as Extended Adjuvant Treatment of Postmenopausal Patients With Primary Breast Cancer.

58. Hellrieqel M, Mueller M, Reimer T, Baerens DT, von der Assen A, Hackmann J, Schmidt K, Baier-Ebert M, Spall T, Emons G (2011) 5201 POSTER The EXpand Study – Effect of Zoledronic Acid on Prevention of Bone Loss, During Extended Adjuvant Therapy With Letrozole in Postmenopausal Women With Primary Hormone Receptor Positive Breast Cancer Compared to Letrozole Alone. Eur J Cancer 47:S390

59. Amir E, Freedman O, Carlsson L, Dranitsaris G, Tomlinson G, Laupacis A, Tannock IF, Clemons M (2013) Randomized feasibility study of de-escalated (Every 12 wk) versus standard (every 3 to 4 wk) intravenous pamidronate in women with low-risk bone metastases from breast cancer. Am J Clin Oncol Cancer Clin Trials 36:436–442

60. Jacobs C, Kuchuk I, Bouganim N, et al (2016) A randomized, double-blind, phase II, exploratory trial evaluating the palliative benefit of either continuing pamidronate or switching to zoledronic acid in patients with high-risk bone metastases from breast cancer. Breast Cancer Res Treat 155:77–84

61. ClinicalTrials.gov S0307 Phase III Trial of Bisphosphonates as Adjuvant Therapy for Primary Breast Cancer. https://clinicaltrials.gov/ct2/show/results/NCT00127205.

62. Charehbili A, van de Ven S, Smit VTHBM, et al (2014) Addition of zoledronic acid to neoadjuvant chemotherapy does not enhance tumor response in patients with HER2-negative stage II/III breast cancer: the NEOZOTAC trial (BOOG 2010-01). Ann Oncol 25:998–1004

63. Lelièvre L, Clézardin P, Magaud L, Roche L, Tubiana-Mathieu N, Tigaud JD, Topart D, Raban N, Mouret-Reynier MA, Mathevet P (2018) Comparative Study of Neoadjuvant Chemotherapy With and Without Zometa for Management of Locally Advanced Breast Cancer With Serum VEGF as Primary Endpoint: The NEOZOL Study. Clin Breast Cancer 18:e1311–e1321

64. Hasegawa Y, Tanino H, Horiguchi J, et al (2015) Randomized controlled trial of zoledronic acid plus chemotherapy versus chemotherapy alone as neoadjuvant treatment of HER2-negative primary breast cancer (JONIE Study). PLoS One 10:1–10

65. ClinicalTrials.gov Efficacy and Safety of Letrozole vs. Letrozole Plus Zoledronic Acid as Endocrine Therapy Before Surgery in Postmenopausal Patients With Breast Cancer (FEMZONE).

66. Novartis (2007) Randomized multi-centre study comparing prolonged primary systemic endocrine therapy with letrozole (Femara*) alone or in combination with zoledronic acid (Zometa*) in early breast cancer (NEOadjuvant Study in CANada). Clin Trial Results Website 1–9

67. Aft R, Chavez-MacGregor M, Trinkaus K, Naughton M, Weilbaecher K (2007) Effect of zoledronic acid on bone loss in women undergoing chemotherapy for breast cancer. Breast Cancer Res Treat 106:S38

68. Dias S, Welton N, Sutton A, Ades A (2011) Technical Support Document 2 : A Generalised Linear Modelling Framework for Pairwise and Network Meta-Analysis of Randomised Controlled Trials . ( Technical Support Document in Evidence Synthesis ; No . TSD2 ). National Institute for Health and Clinical E.

69. Turner RM, Davey J, Clarke MJ, Thompson SG, Higgins JP (2012) Predicting the extent of heterogeneity in meta-analysis, using empirical data from the Cochrane Database of Systematic Reviews. Int J Epidemiol 41:818–827

70. Plummer M (2003) DSC 2003 Working Papers JAGS: A program for analysis of Bayesian graphical models using Gibbs sampling.

71. Van Valkenhoef G, Maintainer JK (2016) Package “gemtc” Title Network Meta-Analysis Using Bayesian Methods. https://doi.org/10.1002/sim.3767

72. van Valkenhoef G, Dias S, Ades AE, Welton NJ (2016) Automated generation of node-splitting models for assessment of inconsistency in network meta-analysis. Res Synth Methods 7:80–93

73. Black DM, Cummings SR, Karpf DB, et al (1996) Randomised trial of effect of alendronate on risk of fracture in women with existing vertebral fractures. Lancet 348:1535–1541

74. Cummings SR, Black DM, Thompson DE, et al (1998) Effect of alendronate on risk of fracture in women with low bone density but without vertebral fractures. Results from the fracture intervention trial. J Am Med Assoc 280:2077–2082

75. Reid IR, Gamble GD, Mesenbrink P, Lakatos P, Black DM (2010) Characterization of and risk factors for the acute-phase response after zoledronic acid. J Clin Endocrinol Metab 95:4380–4387

76. Black DM, Delmas PD, Eastell R, et al (2007) Once-Yearly Zoledronic Acid for Treatment of Postmenopausal Osteoporosis. N Engl J Med 356:1809–22

77. Goss PE, Ingle JN, Alés-Martínez JE, et al (2011) Exemestane for breast-cancer prevention in postmenopausal women. N Engl J Med 364:2381–91
