# Supplementary material for: The adverse effects of bisphosphonates in breast cancer: A systematic review and network meta-analysis
Source: PLoS One. 2021 Feb 5;16(2):e0246441. doi: 10.1371/journal.pone.0246441 (PMC7864400; doi:10.1371/journal.pone.0246441)
Supplement: S2 Table — (DOCX) [file pone.0246441.s004.docx]

# The adverse effects of bisphosphonates as adjuvant therapy in breast cancer: a piggy-back systematic review and network meta-analysis

Christopher Jackson, Alexandra LJ Freeman, Zśofia Szlamka, David J Spiegelhalter

## Supporting Information

**S2 Table 2: Results of a fixed effects meta-regression analysis showing adverse effects where the contrast in the treatment effect between results in patients with or without metastatic breast cancer was statistically significant, and the treatment effect on adverse event risk for the higher-risk subgroup was practically significant (odds ratio > 1.5 or risk difference > 2%).**

|  |  | **Total number of events / number of patients, treated vs control** | | **None metastatic** | | **All metastatic** | | **p-value for subgroup contrast** | |
| --- | --- | --- | --- | --- | --- | --- | --- | --- | --- |
|  | **Studies (none/all metastatic)** | **None metastatic** | **All metastatic** | **Odds ratio** | **Risk difference** | **Odds ratio** | **Risk difference** | **OR** | **RD** |
| Cardiac events | 8/2 | 88/4602 vs 49/3752 | 13/400 vs 6/26 | 1.58 (1.09,2.28) | 0.01 (0,0.01) | 0.11 (0.04,0.33) | -0.2  (-0.36,-0.04) | 0.00 | 0.01 |
| Dizziness | 7/3 | 35/2274 vs 15/2277 | 59/514 vs 27/139 | 2.21 (1.2,4.06) | 0.01 (0,0.02) | 0.71 (0.38,1.32) | -0.02  (-0.1,0.05) | 0.01 | 0.41 |
| Fatigue | 13/5 | 1217/4678 vs 1200/4708 | 348/951 vs 170/605 | 1.07 (0.88,1.3) | 0.01  (-0.01,0.02) | 1.69 (1.24,2.29) | 0.11  (0.06,0.17) | 0.01 | 0.00 |
| Hypocalcemia | 2/8 | 3/2062 vs 2/2076 | 149/1530 vs 58/1159 | 1.28 (0.16,10.34) | 0  (-0.02,0.02) | 2.14 (1.19,3.85) | 0.06  (0.03,0.08) | 0.64 | 0.00 |
| Increased bone pain | 11/5 | 355/2982 vs 267/3002 | 76/820 vs 53/453 | 1.53 (1.26,1.85) | 0.02 (0,0.04) | 0.64 (0.39,1.06) | 0  (-0.03,0.03) | 0.00 | 0.18 |
| Influenza-like symptoms | 4/2 | 49/137 vs 25/133 | 16/306 vs 4/314 | 2.86 (0.93,8.82) | 0.22 (0.12,0.32) | 4.28 (0.93,19.63) | 0.04  (-0.01,0.09) | 0.68 | 0.00 |
| Neuralgia | 1/2 | 4/1612 vs 1/1623 | 5/400 vs 2/26 | 4.03 (0.45,36.14) | 0  (0,0) | 0.14 (0.02,0.83) | -0.06  (-0.17,0.04) | 0.02 | 0.21 |
| Neutropenia | 4/3 | 306/3443 vs 341/3474 | 52/514 vs 19/139 | 0.87 (0.71,1.07) | -0.01  (-0.02,0) | 1.04 (0.53,2.05) | 0.05  (-0.01,0.11) | 0.62 | 0.05 |
| Osteonecrosis of the jaw | 13/3 | 23/6536 vs 0/5727 | 4/470 vs 0/106 | 5.04 (1.69,15.07) | 0  (0,0) | 0.34 (0.04,3.01) | 0  (-0.02,0.03) | 0.03 | 0.99 |
