# Supplementary material for: The adverse effects of bisphosphonates in breast cancer: A systematic review and network meta-analysis
Source: PLoS One. 2021 Feb 5;16(2):e0246441. doi: 10.1371/journal.pone.0246441 (PMC7864400; doi:10.1371/journal.pone.0246441)
Supplement: S3 Table — (DOCX) [file pone.0246441.s005.docx]

**S4 Table 3: Calculated absolute risks for some adverse effects (and treatments) where baseline risks were available for women of a similar demographic but without breast cancer (taken from trial NCT00083174[77]).**

| Outcome | Treatment | Odds ratio from  direct comparisons | Odds ratio from network meta-analysis | Number of women of similar demographic without breast cancer who would be expected to experience the outcome | |
| --- | --- | --- | --- | --- | --- |
|  |  |  |  | If they **were NOT** taking the treatment | If they **were** taking the treatment |
| **Back pain** | Nitrogenous bisphosphonates | 1.54  (1, 2.37) | 1.64 (1.08, 2.69) | **10**  out of 100 | **15**  (11-23) out of 100 |
| **Cardiac events**  (excluding congestive heart failure) | Nitrogenous bisphosphonates | 2.51  (1.52, 4.17) | 2.73 (1.33, 6.31) | **0.2**  out of 100 | **0.2**  (0.2-0.3) out of 100 |
|  | Non-nitrogenous bisphosphonates (clodronate) | 5.22  (0.25, 109.67) | 4.35 (1.44, 13.6) | **0.2**  out of 100 | **0.3**  (0.2-0.4) out of 100 |
| **Nausea *** | Any bisphosphonate | 1.21  (1.08, 1.35) | 1.33  (1.11, 1.63) | **5**  out of 100 | **7**  (6-9) out of 100 |
| **Diarrhoea** | Oral clodronate (also possibly true for others taken orally) | 3.34  (1.18, 9.45) | 1.81 (1.35, 2.74) | **3**  out of 100 | **6**  (5-9) out of 100 |
| **Thrombo-embolic events** | Zoledronic acid | 3.18  (1.34, 7.51) | 2.74 (0.98, 7.65) | **0.1**  out of 100 | **0.1**  (0.1-0.11) out of 100 |
|  | Ibandronate |  | 13.33  (2.43, 73.09) | **0.1**  out of 100 | **0.1**  (0.1-0.16) out of 100 |
| ***** | Clodronate |  | 7.27  (1.41, 33.44) | **0.1**  out of 100 | **0.1**  (0.1-0.13) out of 100 |
| **Fatigue** | Any bisphosphonate | 1.13  (1.02, 1.26) | 1.28  (1.06, 1.65) | **21**  out of 100 | **25**  (22-30) out of 100 |

Note that these baseline risks were only available for a subset of the adverse effects of bisphosphonates identified by our meta-analysis (please see main text Table 2 for the complete list).

** Finding not upheld when analysis run on only 24 trials with comprehensive adverse event reporting (reporting threshold <5% of patients suffering the event)*

*Colours represent quality of evidence (green=highest, yellow=lower, red=lowest, based on replicability of results between full dataset and comprehensive reporting subset, and number of patients included in direct comparison trials)*
