# Supplementary material for: The adverse effects of bisphosphonates in breast cancer: A systematic review and network meta-analysis
Source: PLoS One. 2021 Feb 5;16(2):e0246441. doi: 10.1371/journal.pone.0246441 (PMC7864400; doi:10.1371/journal.pone.0246441)
Supplement: S1 File — (DOCX) [file pone.0246441.s006.docx]

# The adverse effects of bisphosphonates as adjuvant therapy in breast cancer: a piggy-back systematic review and network meta-analysis

Christopher Jackson, Alexandra LJ Freeman, Zśofia Szlamka, David J Spiegelhalter

## Supporting Information

## Network meta-analysis methods

The standard Bayesian random-effects meta-analysis model for a binary outcome was used[1]. The extent of heterogeneity between studies (random effects variance) is assumed to be the same for all treatment comparisons for a particular event, allowing the standard adjustment for correlation between different comparisons from studies with three or more arms[1]. Informative prior distributions for these variances were used, specifically the distributions derived by Turner et al.[2] from the Cochrane database of published meta-analyses of comparisons of pharmaceutical interventions for “semi-objective” outcomes. Independent *t* prior distributions are used for each log odds ratio, with mean 0, 1 degree of freedom, and a precision parameter of 2, implying a prior 95% credible interval of (0.04, 24) for the odds ratio. Markov Chain Monte Carlo simulation was used to fit the models, using the JAGS software[3] and the *gemtc* R package[4] modified to allow groups of similar treatments to have the same effect.

For the best-fitting treatment classification, the treatment effect on the risk of symptoms is reported as a pooled odds ratio, representing the expected odds ratio in an “average” study population. The equivalent pooled odds ratio from direct evidence only is reported for comparison. “Node-splitting” methods[5] were used to check for consistency between direct and indirect evidence, for the symptoms and treatments for which both direct and indirect evidence were available. Essentially, the network is split into two components, one providing only direct and the other providing only indirect evidence on the treatment effect. The ratio between the treatment effect (odds ratio) obtained from direct evidence only, and the effect from indirect evidence only, gives a measure of evidence consistency.

## Network meta-analysis model comparison

We illustrate the network meta-analysis procedure for the common event of myalgia in S3 Fig 2a-d. S3 Fig 2a shows network diagrams indicating the number of studies making each direct comparison of rates of myalgia between different pairs of treatments, on the left, distinguishing drug treatments by their doses and delivery methods, and on the right, distinguishing only different drugs. The best-fitting model, according to the deviance information criterion, distinguished different drugs but not doses or delivery methods. The majority of evidence for this adverse event came from 14 direct comparisons of zoledronic acid with a non-bisphosphonate control. S3 Fig 2b shows the raw data from each of these studies. In several study arms, more than 40% of participants suffered myalgia, though many study arms reported less than 10%. Looking at those with low reported rates, NEOZOTAC & NSABP B-34 only reported ‘serious’ adverse events, explaining their low percentages. (ProBone 1 was a tiny study with only 1 case of myalgia amongst its 11 participants, which happened to be a control participant, explaining its ‘reversed’ pattern).

From this model (S3 Figure 2c) we judge there is a strong and significant risk of myalgia associated with zoledronic acid, the estimated effects of pamidronate and ibandronate are similar but the evidence is weaker, and there was no risk associated with clodronate. The direct evidence informing the effect of zoledronic acid suggests a slightly smaller, but still substantial, effect. S3 Figure 2c further breaks down the evidence behind the best-fitting model, showing in red all study-specific direct comparisons of bisphosphonates against observation-only or placebo: we note there is considerable between-study heterogeneity in odds ratios, although less than that of the baseline rates. The pooled odds ratio from a meta-analysis of the direct comparisons against observation-only is slightly lower than the estimate from network meta-analysis, showing the influence of the indirect evidence.


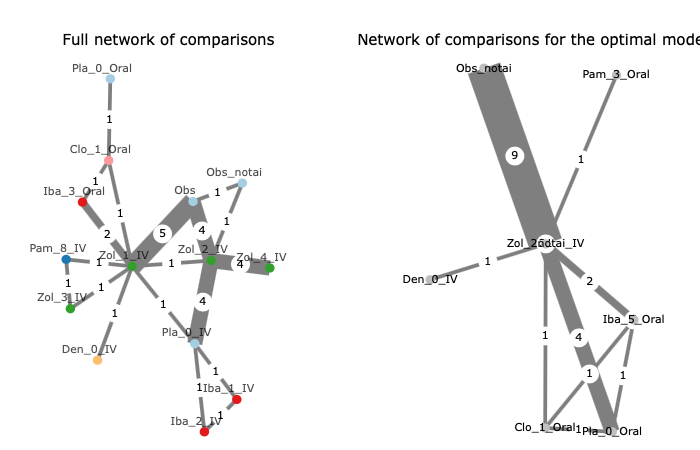


**S3 Fig 2a. Full network diagrams for the outcome of myalgia.** On the left hand side, network diagram showing the full network of comparisons available across all trials reporting data on an outcome of myalgia (each drug and dose treated separately), and on the right, the network of comparisons for the best-fitting network meta-analysis model which distinguishes drugs but considers all doses to have the same effect on the risk of an adverse event. Treatments are labelled by their dosage and delivery method (key below).

Numbers indicate dosages:

| Zol | Zoledronic acid |
| --- | --- |
| 1 | 4 or 5mg every 3 or 4 weeks |
| 2 | 4mg every 3 or 6 months |
| 3 | 8mg every 3 or 4 weeks |
| 4 | ‘delayed’ (4mg every 3-4 weeks once patients show low bone mass) |
| Pam…IV | Pamidronate (iv) |
| 1 | 45mg every 3-4 weeks |
| 2 | 12mg start then oral 50mg daily |
| 4 | 30mg per day for 2 weeks, then 10 weeks without |
| 5 | 30mg every 2 weeks |
| 6 | 60mg every 4 weeks |
| 7 | 60mg every 2 weeks |
| 8 | 90mg every 4 weeks |
| 9 | 60mg every 3 weeks |
| Pam…Oral | Pamidronate (oral) |
| 2 | 300mg daily |
| 3 | 200 mg daily |
| Iba | Ibandronate |
| 1 | 2mg every 3-4 weeks |
| 2 | 6mg every 3-4 weeks |
| 3 | 50mg daily (oral) |
| 4 | 12mg iv start then 50mg oral daily |
| 5 | 150mg every 4 weeks |
| 6 | 20mg daily (oral) |
| Clo | Clodronate |
| 1 | 1600mg oral daily |
| 2 | 2400mg oral daily |
| 3 | 900mg iv every 3 weeks |
| 4 | 300mg iv daily |
| Pla | Placebo |
| Obs | Observation only |
| Obs_notai | Observation only (arm in which patients were not receiving aromatase inhibitors) |
| Den | Denosumab |


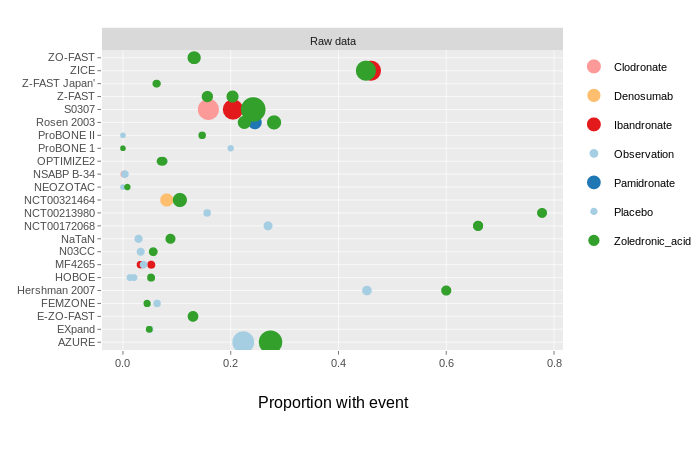


**S3 Fig 2b. Raw data from those trials reporting myalgia, showing the proportion of patients suffering from myalgia.** Size of blob indicates number of patients, colour of blob indicates treatment group they were in.


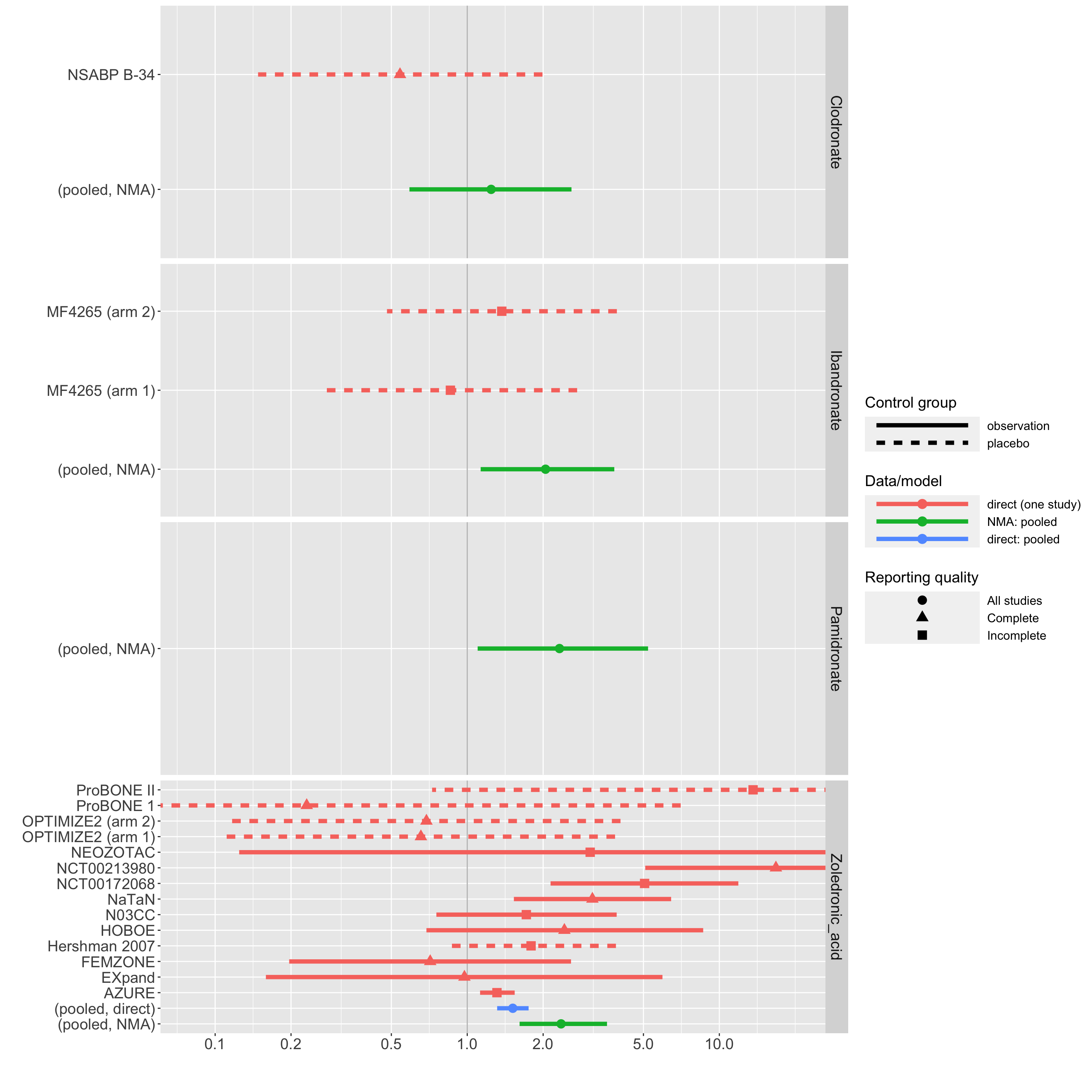


**S3 Fig 2c. Detailed network meta-analysis results for the adverse effect of myalgia, under the best fitting network meta-analysis model for this event, which distinguishes the effects of four different drugs.** Each of the panels shows in green the network meta-analysis pooled odds ratios of myalgia for the four respective drugs. Where there is evidence from studies that directly compared this drug with a non-bisphosphonate control, this is also shown as red lines. Solid lines are comparisons against an observation-only control, and dotted lines are comparisons with a placebo group. Where there are comparisons with observation-only, the fixed effects meta-analysis pooled estimate is shown in blue.

S3 Fig 3 compares the results of the five network meta-analysis models that were fitted for the adverse event of myalgia, each one with a different classification of bisphosphonates. Each line is an estimate of the odds ratio of myalgia comparing a particular bisphosphonate treatment with observation only. The best-fitting model, according to the deviance information criterion, distinguished different drugs but not doses or delivery methods (fourth panel down).


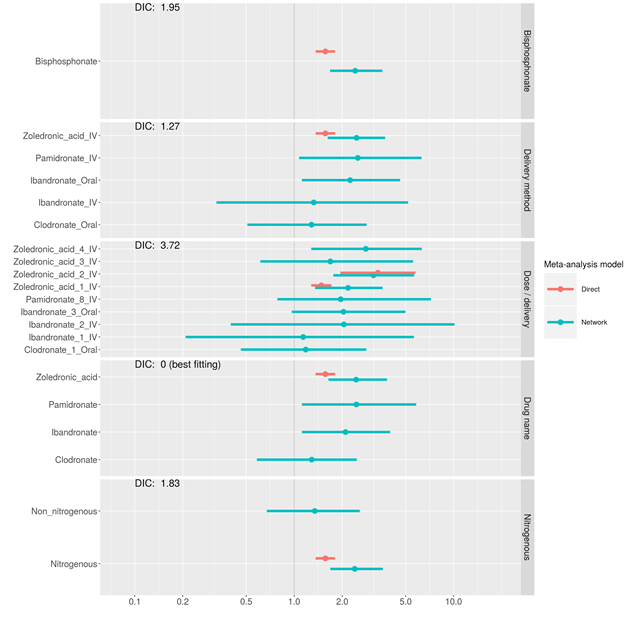


**S3 Fig 3. Comparison of five alternative network meta-analysis models for the outcome of myalgia.** Each of the five panels shows pooled odds ratios of myalgia for a bisphosphonate treatment compared to observation-only, under a different classification of bisphosphonates. Where direct data are available for the treatment comparison, the pooled odds ratio from a fixed effects direct data meta-analysis is also shown in red, alongside network meta-analysis pooled estimates in blue. The deviance information criterion (DIC) relative to the best-fitting model is shown on the top of each panel. The best fitting model has a DIC of 0, higher DICs indicate worse fit, DIC differences of about 2 or less are considered small, and “not converged” (middle panel) indicates that there was insufficient information for the estimates to be reliable. In this case the best fitting model considers each different named drug to have a different effect on the risk of myalgia, while doses and delivery methods of the same drug have the same effect.


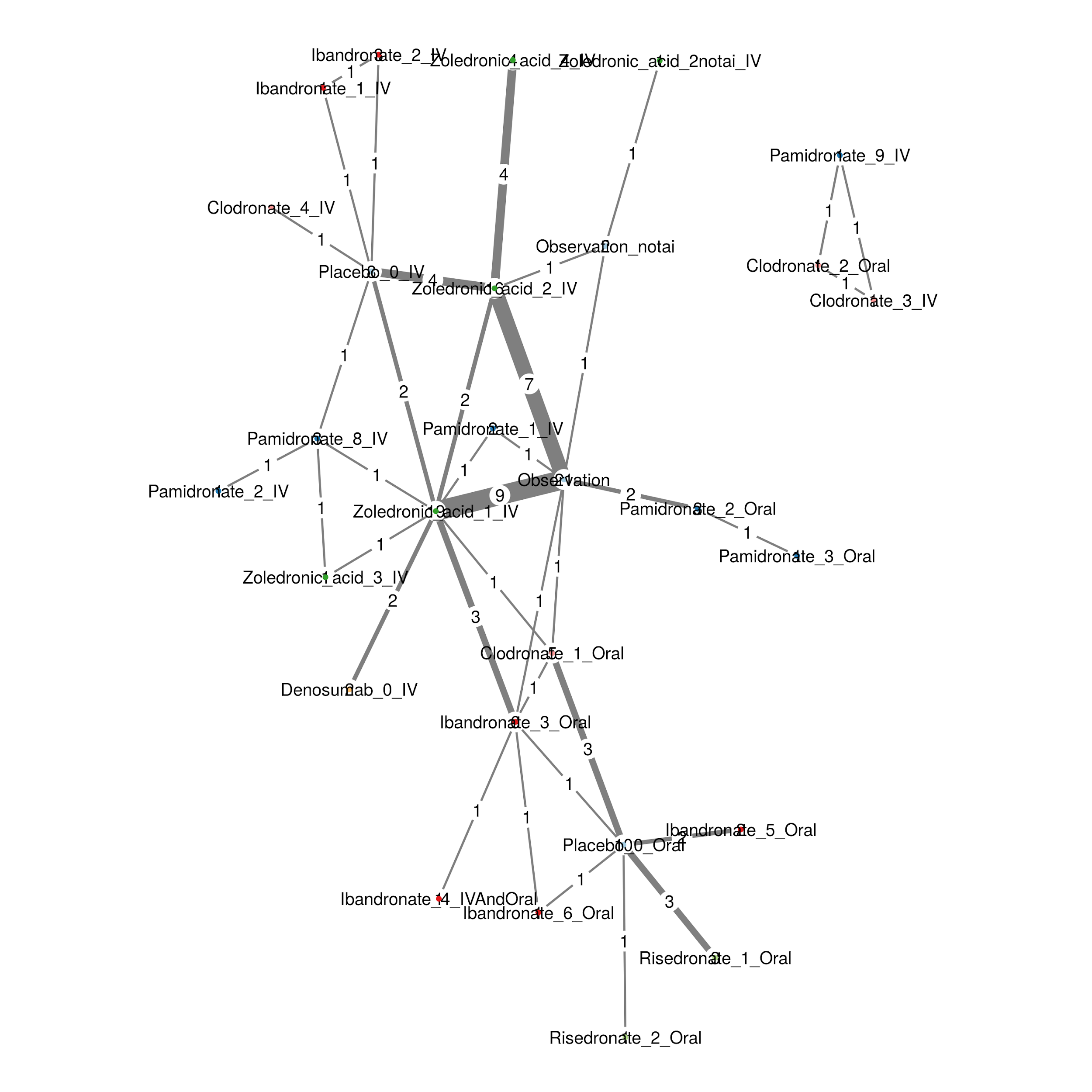


**S3 Fig 4.** **Network diagram of the trials included in the meta-analysis, showing the number of trials making each comparison.** This shows the most fine-grained breakdown of the different drugs tested (separating them by drug, by dose – coded by numbers (see below) – and by delivery method – intravenous (IV) or oral. The points in the diagram represent different treatments, as defined by the finest-grain subcategorization, separating individual bisphosphonate drugs (pamidronate, ibandronate, clodronate, risedronate and zoledronic acid), and the different control arms (denosumab – a monoclonal antibody tested as an alternative to bisphosphonate in some trials; observation only). “notai” indicates the arm was distinguished by using a hormone therapy that is not an aromatase inhibitor, where the other study arms used an aromatase inhibitor).

Numbers indicate dosages:

| Zoledronic acid |  |
| --- | --- |
| 1 | 4 or 5mg every 3 or 4 weeks |
| 2 | 4mg every 3 or 6 months |
| 3 | 8mg every 3 or 4 weeks |
| 4 | ‘delayed’ (4mg every 3-4 weeks once patients show low bone mass) |
| Pamidronate (iv) |  |
| 1 | 45mg every 3-4 weeks |
| 2 | 12mg start then oral 50mg daily |
| 4 | 30mg per day for 2 weeks, then 10 weeks without |
| 5 | 30mg every 2 weeks |
| 6 | 60mg every 4 weeks |
| 7 | 60mg every 2 weeks |
| 8 | 90mg every 4 weeks |
| 9 | 60mg every 3 weeks |
| Pamidronate (oral) |  |
| 2 | 300mg daily |
| 3 | 200 mg daily |
| Ibandronate |  |
| 1 | 2mg every 3-4 weeks |
| 2 | 6mg every 3-4 weeks |
| 3 | 50mg daily (oral) |
| 4 | 12mg iv start then 50mg oral daily |
| 5 | 150mg every 4 weeks |
| 6 | 20mg daily (oral) |
| Risedronate |  |
| 1 | 35mg per week |
| 2 | 30mg per day for 2 weeks, 10 weeks without |
| Clodronate |  |
| 1 | 1600mg oral daily |
| 2 | 2400mg oral daily |
| 3 | 900mg iv every 3 weeks |
| 4 | 300mg iv daily |

1. Dias S, Welton N, Sutton A, Ades A. 2011 Technical Support Document 2 : A Generalised Linear Modelling Framework for Pairwise and Network Meta-Analysis of Randomised Controlled Trials . ( Technical Support Document in Evidence Synthesis ; No . TSD2 ). National Institute for Health and Clinical E.

2. Turner RM, Davey J, Clarke MJ, Thompson SG, Higgins JP. 2012 Predicting the extent of heterogeneity in meta-analysis, using empirical data from the Cochrane Database of Systematic Reviews. *Int. J. Epidemiol.* **41**, 818–827. (doi:10.1093/ije/dys041)

3. Plummer M. 2003 DSC 2003 Working Papers JAGS: A program for analysis of Bayesian graphical models using Gibbs sampling. *DSC 2003 Work. Pap.*

4. Van Valkenhoef G, Maintainer JK. 2016 Package ‘gemtc’ Title Network Meta-Analysis Using Bayesian Methods. (doi:10.1002/sim.3767)

5. van Valkenhoef G, Dias S, Ades AE, Welton NJ. 2016 Automated generation of node-splitting models for assessment of inconsistency in network meta-analysis. *Res. Synth. Methods* **7**, 80–93. (doi:10.1002/jrsm.1167)
