# Supplementary material for: The adverse effects of bisphosphonates in breast cancer: A systematic review and network meta-analysis
Source: PLoS One. 2021 Feb 5;16(2):e0246441. doi: 10.1371/journal.pone.0246441 (PMC7864400; doi:10.1371/journal.pone.0246441)
Supplement: Attachment — Submitted filename: Response to Reviewers.docx [file pone.0246441.s007.docx]

8^th^ June 2020,

Dear Editor,

We here outline our responses to the three reviewers to our manuscript PMEDICINE-D-20-00800R2.

___________________________________________________________________________

Reviewer #1: The authors have addressed all my comments thoroughly

Michael Dewey

Thank you!
___________________________________________________________________________

Reviewer #2: The revised manuscript has clarified a number of points but remains verbose, wandering and at points awkward. The following points outline examples of where these criticisms are raised, but is not an all-inclusive list.

* There is no need for the introduction to outline that the authors spoke to clinicians and patients or that a series of reviews and meta-analyses were undertaken. This type of conversational text is throughout the manuscript and can be trimmed.

We have shortened this phrasing, whilst retaining the evidence of patient involvement in the initiation of the study.

We have also further reduced verbage throughout the manuscript and increased use of the passive voice.

* The manuscript does not specify what is intend by the term "adjuvant" for the purpose of this study. Typically, "adjuvant" refers to an anticancer therapy for women with breast cancer, yet many of the RTCs analyzed are studies of a bisphosphonate used for the treatment or prevention of osteoporosis (not cancer).  Indeed, there are studies of metastatic disease included as well. Metastatic is not adjuvant! This blurring of clinical situations is a significant weakness to the manuscript.

We thank the reviewer for pointing this lack of clarity out. Although our initial aim had been adjuvant use only, we found that incorporating the data from trials in metastatic cancer was statistically valid and increased the power of the study considerably. Since the results are shown to be valid both for adjuvant use in early breast cancer and for treatment in women with metastatic cancer we have adjusted the title and the wording of the manuscript throughout to reflect this.

* Page 7 outlines 4 questions but does not tie them into specific aims/objectives/endpoints or use the questions to structure the manuscript's Introduction, Methods, Results or Discussion. This further adds to the sense of rambling, conversational presentation.

We have now used those 4 questions overtly in the Introduction, Methods and the Results (restructuring slightly to accommodate them). Although this increases the word count, it does hopefully show the relevance of each part of the manuscript to answering the key questions/aims.

The Discussion already starts with a summary of the key aims using slightly different language, and so we have preferred to keep this format. Some of the key questions (such as ‘what is the likely severity of those adverse events?’) are only answered in Table 2, so it seemed inappropriate to raise them all overtly again as sections within the Discussion.

* The Methods, page 9, state that the "most recent papers published" were used to extract the adverse events, yet the safety / toxicity reporting may have been more detailed in an earlier publication. More information on how serial publications were managed and from where the toxicity data was abstracted should be provided

We have updated the text describing the method to read “Data were extracted from a trials registry website when possible. If results were not published in a trials registry, all published papers regarding a trial were examined for adverse data, and data was taken from the most recent paper that included adverse data (i.e. with the longest follow-up time possible).”

* Page 10 states that data was collected on whether the women had bone metastases. The presence of any metastases does not belong in an adjuvant analysis.

As above, we have adjusted the title and introduction to reflect the fact that the study incorporates use bisphosphonates in either an adjuvant or metastatic setting.

The Supplemental materials demonstrate that studies of patients with bone metastases were included in the analysis (for example: supplement references 29 and 52 are studies enrolling patients with metastatic disease).

Indeed, we have now included (lines 327-335; 455-468 and Supplementary Table 2) the data on the direct meta-analysis including cancer stage as a regression term.

* Page 11 states that the bisphosphonate dosing used in AZURE is not common in clinical use. This statement needs a reference.

We have now added this reference (Coleman et al, 2011).

* The inclusion of the Horizon and Fit trials is awkward. This analysis is not included in one of the four key questions. Throwing in another analysis adds to the rambling character of the manuscript.

We have removed the extra analysis of these two trials.

Rather than "cherry pick" two osteoporosis studies to identify expected toxicities, it would be more widely applicable to compare to the toxicities outlined in the packet insert of each bisphosphonate.

This is an excellent suggestion and we have now incorporated a list of the side effects listed for bisphosphonates in the British National Formulary (lines 166-171) and mentioned a comparison with their list in our Discussion (lines 735-737).

* The patient level data analysis of the Azure study could be removed.  The Authors state that the Azure zoledronic acid dosing/interval regimen is not used clinically - making this analysis even less interesting). The Azure data adds bulk to the manuscript. Consider moving the Azure data to a separate manuscript.

We have adjusted the wording to make clear why the AZURE data was suitable for the purpose of investigating the potential for interactions between bisphosphonates and other concurrent therapies. Coleman et al. 2011 state: “combining ZOL with chemotherapy has the potential for enhanced toxicity, especially if there is a synergistic interaction with chemotherapy on normal tissues. Safety evaluation within the AZURE trial is the ideal

opportunity to assess this. Here, we report the largest data set evaluating safety of ZOL outside the metastatic setting and the first analysis at this intensive dosing schedule addressing specific adverse events of note”.

Without the analysis of individual-level data we would be unable to assess the possibility of synergistic interactions in toxicity, one of our key stated aims. (We had already demonstrated the lack of a significant effect of dosing schedule on adverse event profile, and the results from the AZURE trials are demonstrably in line with those from the other trials in the meta-analysis, which we have also now made clear in the manuscript).

o The existing reference list permits for subsetting premenopausal from postmenopausal women. Note that ABCSG12, Hershman, CALGB79809, Hoboe, etc specifically enrolled premenopausal women, while other studies restricted enrollment to postmenpausal women.  The Authors are able to examine an element of demographic data within the studies identified for the primary analysis.

We were indeed able to and have now included (lines 327-335; 444-453) the data on the direct meta-analysis including menopausal status as a regression term.

* Page 36 mentions osteonecrosis related to denosumab, but the Methods do not outline the procedures by which such denosumab data was obtained. The Authors should not drop in findings unassociated with specific Aims/Objectives/Endpoints and the Methods used. This is another example of the manuscript lacking a crisp focus.

As lines 177 and 285 describe, in the methods, denosumab was sometimes used as a comparison arm in trials, and was therefore used as a potential ‘control’ group (allowing greater use of indirect data in the network meta-analysis by joining networks). Line 413 in the Results section again mentions this.

However, we have removed the mention of the number of cases of ONJ reported in the denosumab arms from the Discussion as the Reviewer is right to point out that it is not related to the aims of this study.

Reviewer #3: The authors have adequately addressed the questions raised by reviewers and the Editor. The paper can now be accepted. Important work! Michael Gnant

Thank you!
