# Supplementary material for: The adverse effects of bisphosphonates in breast cancer: A systematic review and network meta-analysis
Source: PLoS One. 2021 Feb 5;16(2):e0246441. doi: 10.1371/journal.pone.0246441 (PMC7864400; doi:10.1371/journal.pone.0246441)
Supplement: Attachment — Submitted filename: review for Plus One.docx [file pone.0246441.s008.docx]

Dear editors,

The manuscript entitled “The adverse effects of bisphosphonates in breast cancer: a systematic review and network meta-analysis” describe through and comprehensive analyses of the toxicity associated with bisphosphonates in breast cancer, both in the adjuvant and in the advanced setting. Quantification of the risks from bisphosphonates is of value and may better clarify to the risk and benefit balance when discussing with the patient treatment options. While it seems that the authors have invested many efforts and hard work for this study, there several important limitations to their work.

Major limitations:

1. “Data were extracted from a trials registry website when possible. If results were not published in a trials registry, all published papers regarding a trial were examined for adverse data, and data was taken from the most recent paper that included adverse data”. Data reported in the registry website often lacking or inaccurate (BMJ Open. 2017 Oct 11;7(10):e017719.  doi: 10.1136/bmjopen-2017-017719) The most acceptable method in meta-analyses to use either the data reported in the manuscripts or to conduct patient-level data. This is a major limitation of this study.
2. “When ‘serious’ and ‘non serious’ events were reported (for example, as on clinicaltrials.gov), these were recorded as Grades 3/4 and Grades 1/2 respectively.” This use is incorrect at the definition of serious AE is different from the grading of CTCAE.
3. Results of studies comparing bisphosphonates to placebo/ no treatment should to be pooled together with studies comparing bisphosphonates to other treatment (such as denosumab).

Additional comments:

1. Generally, the text thought its sections is too long and not concise. This has negative effect on the readability of the manuscript. Specifically, the abstract should be more concise and should be shorten (currently 500 words). Description of the results should be shorter and more concise. In the results sections there are descriptions of the rational to preform and describe the analyses- some of these descriptions do not improve the clarity of the text and can be omitted, others should be moved to the methods section.
2. In the last paragraph of the introduction (row 173) details of the search should not be elaborated (this should be in the methods section), only concise description for the purpose of the study and the rationale for conduction the study.
4. Row 181-184 should be deleted.
5. Classification to early/ metastatic
6. Numbering of figures should be edited by the order it is mentioned in the manuscript
7. Figure 1-it is not clear how many studies were included. In the text box in the bottom of the figure- there is 56 studies. Do the text-boxes from the left to this text box describe additional studies?
8. I could not find figure 2 in the text.
9. Along to text there are preceding questions- to improve the readability of the manuscript, I suggest to omit these questions.
10. For the subgroup analysis in the AZURE study- could the interaction between the subgroup for the significant findings (arthritis) could be calculated and presented? Also, for the forest plots- could the size of the square or the circle be in proportion to the number of patients in each group?
11. For figure 4- can interaction for between the treatment options be presented for the different adverse event. According to the text fever and headaches showed significant interaction, but by the figure, additional adverse events might be influenced as well by the type of treatment
